# Whole-exome sequencing in Saudi colorectal cancer patients reveals distinct mutational patterns and population specific pathogenic variants

**DOI:** 10.3389/fonc.2025.1679528

**Published:** 2025-10-08

**Authors:** Hanan E. Alatwi, Amnah A. Alharbi, Rashid Mir, Othman R. Alzahrani, Abdulrahman H. Alessa, Yousef M. Hawsawi, Mohammed Ali Arishi, Aziz Dhaher Albalawi

**Affiliations:** ^1^ Department of Biology, Faculty of Science, University of Tabuk, Tabuk, Saudi Arabia; ^2^ Department of Biochemistry, Faculty of Science, University of Tabuk, Tabuk, Saudi Arabia; ^3^ Department of Medical Lab Technology, Faculty of Applied Medical Sciences, and Prince Fahd Sultan Research Chair for Biomedical Research, University of Tabuk, Tabuk, Saudi Arabia; ^4^ Research Center, King Faisal Specialist Hospital and Research Center, Jeddah, Saudi Arabia; ^5^ Department of Biochemistry and Molecular Medicine, College of Medicine, Alfaisal University, Riyadh, Saudi Arabia; ^6^ Faculty of Laboratory, King Khaled Hospital, Ministry of Health, Tabuk, Saudi Arabia

**Keywords:** colorectal cancer, Saudi cohort, whole-exome sequencing, somatic mutation, pathway analysis, precision oncology

## Abstract

**Background:**

Colorectal cancer (CRC) shows significant inter-population heterogeneity in its genomic landscape, yet Middle Eastern populations are underrepresented in large-scale sequencing studies. This exploratory study aims to characterize somatic mutations and disrupted signaling pathways in Saudi Arabian CRC patients.

**Methods:**

We performed whole-exome sequencing (WES) on tumor DNA from 24 Saudi CRC patients. Somatic variants were identified and analyzed in a curated panel of cancer-related genes. Comparative analysis was conducted against The Cancer Genome Atlas colorectal cancer dataset (TCGA-COADREAD), and pathway enrichment analysis was performed.

**Results:**

Somatic variants were identified in 23 tumors, with recurrent mutations in *BRCA2* (61%), *TCF7L2* (52%), *EGFR* (43%), and *SOS1* (43%). Compared to TCGA-COADREAD, mutation frequencies were significantly higher in *BRCA2*, *EGFR*, *SLC25A5*, and *PIK3R2* (adjusted p < 0.0001). Among 258 total variants, 43% were novel, and 25 were classified as pathogenic, likely pathogenic, or deleterious, including 13 novel variants across nine genes. Pathway analysis revealed frequent disruptions in WNT/β-catenin (65%), homologous recombination (61%), PI3K (48%), and RTK/RAS (43%) signaling pathways.

**Conclusion:**

Our results reveal a distinct mutational profile in Saudi CRC patients, characterized by novel and enriched somatic variants affecting key oncogenic pathways. These findings underscore the necessity of including underrepresented populations in cancer genomics to support globally equitable precision oncology.

## 1 Introduction

Colorectal cancer (CRC) is one of the most prevalent malignancies worldwide, ranking third in incidence and second in cancer-related mortality (1). While early detection and treatment programs in high-income countries have led to a decline in both incidence and mortality, CRC continues to pose a significant global health challenge. In contrast, many low- and middle-income countries, including those in the Arab region, are witnessing a steady rise in CRC incidence. Within the Gulf Cooperation Council (GCC), CRC is the second most common cancer among both men and women. In Saudi Arabia specifically, it accounts for approximately 10.1% of male and 9.3% of female cancer diagnoses (2, 3). These figures underscore the urgent need for region-specific research to better understand the molecular characteristics of CRC in Arab populations.

Large-scale genomic initiatives such as The Cancer Genome Atlas (TCGA) have identified key driver mutations, particularly in genes like *APC*, *KRAS*, *TP53*, and *PIK3CA*, that have shaped our understanding of CRC pathogenesis (4, 5). However, these studies predominantly involve Western populations, limiting their generalizability to more genetically diverse groups. Since genetic variability can influence cancer susceptibility, progression, and treatment response, population-specific genomic studies are essential for advancing precision oncology tailored to regional contexts.

CRC development is driven by the stepwise accumulation of genetic and epigenetic alterations affecting oncogenes, tumor suppressor genes, and DNA repair mechanisms. Key underlying processes include chromosomal instability (CIN), microsatellite instability (MSI), and the CpG island methylator phenotype (CIMP) (6, 7). The advent of high-throughput sequencing technologies, especially whole-exome sequencing (WES), now enables comprehensive profiling of somatic mutations, facilitating the identification of both well-known alterations and novel, population-specific variants (8). A recent Saudi-based study using targeted next-generation sequencing (NGS) reported high mutation frequencies in *BRCA2*, *CHEK1*, *ATM*, and *PMS2*, pointing to a potentially unique mutational landscape in this population. Although the study did not include some canonical CRC genes and found limited clinical correlations, it highlighted the critical role of molecular profiling in guiding personalized treatment strategies (2). Our previous studies have identified novel SNPs in *Toll-like receptor 2* (*TLR2*) (9) and variants in the *HER1* and *HER2* genes (10) that associated with CRC in Saudi population.

In this context, the present study aimed to explore a targeted genomic analysis of CRC in a Saudi Arabian cohort using WES. The focus was on a curated panel of cancer-associated genes, encompassing well-established CRC drivers, *BRCA2*, *EGFR*, *PIK3R2*, *PTEN*, *AXIN1*, *TGFB2*, *SOS1*, *BAX*, and *TCF7L2*, as well as novel or understudied genes identified from our preliminary cohort-specific analyses. These established genes are involved in key signaling pathways such as Wnt/β-catenin, PI3K/AKT/mTOR, and MAPK, all of which are commonly dysregulated in CRC and contribute to tumorigenesis through impaired proliferation, apoptosis, and genomic stability (4, 8, 11–13).

In addition, the study explored a subset of novel or less-characterized genes, including *KRT8*, *KRT18*, *TUBB6*, *SLC25A5*, *ELAVL1*, and *PRDX1*, that were frequently mutated in this cohort. Though not traditionally associated with CRC, these genes may play important roles in tumor biology. For instance, *KRT8* and *KRT18* are cytoskeletal proteins that enhance motility and invasiveness in epithelial cancers (14–16), while *PRDX1* is involved in redox regulation and may contribute to chemoresistance (17). TUBB6, a beta-tubulin isotype, is crucial for microtubule function during cell division, and its disruption may drive chromosomal instability, a hallmark of CRC (18, 19). *SLC25A5* (also known as *ANT2*) facilitates ATP transport across the mitochondrial membrane and has been shown to reduce proliferation and promote apoptosis in colon cancer cells, partly through inhibition of MAPK signaling (20). Finally, *ELAVL1*, an RNA-binding protein that stabilizes mRNAs linked to cell growth and survival, is upregulated in multiple cancer types and has been implicated in tumor progression (21–24).

## 2 Materials and methods

### 2.1 Study population and ethical approval

This study included a cohort of 24 patients with clinically and pathologically confirmed CRC. Tissue specimens were collected from formalin-fixed paraffin-embedded (FFPE) blocks archived at the Division of Histopathology, King Khaled Hospitals, Tabuk, Saudi Arabia. Due to challenges in obtaining matched normal tissue samples from these archived FFPE blocks and existing budgetary constraints, this study adopted a tumor-only whole-exome sequencing approach for somatic variant discovery. All tumor specimens were collected at the time of diagnostic surgery, and none of the patients had received chemotherapy, radiotherapy, or targeted therapy before sample collection.

The research protocol was reviewed and approved by the Institutional Ethical Committee of the University of Tabuk (Protocol No. UT-115-13-2020). All procedures involving human participants were conducted in accordance with the ethical standards of the institutional and national research committees and with the 2013 revision of the Declaration of Helsinki.

#### 2.1.1 Inclusion and exclusion criteria

Participants were selected based on the following inclusion criteria: a confirmed diagnosis of CRC through clinical, histopathological, and radiological assessments; Saudi Arabian nationality; and disease at any clinical stage. While the ethics-approved inclusion criteria permitted enrolment of patients with prior chemotherapy, radiotherapy, or hormone therapy, in practice, all patients included in this study were treatment-naïve at the time of tissue collection.

Exclusion criteria included: non-Saudi nationals, individuals of non-Arab descent, newly naturalized citizens, and patients presenting with multiple primary tumors. Patients unable to adhere to the study requirements or complete the consent process were also excluded. This ethnically focused recruitment strategy was designed to investigate population-specific genomic alterations in CRC.

#### 2.1.2 Data collection

A standardized questionnaire was administered to each patient to collect demographic data, family medical history, and prior awareness of colorectal cancer. Additional clinical and laboratory information was obtained from medical records to create a comprehensive dataset for each participant. Informed consent was obtained in writing from all patients before inclusion, in compliance with the ethical guidelines of the University of Tabuk’s Research Ethics Committee.

### 2.2 DNA extraction, library preparation, and whole-exome sequencing

Genomic DNA was extracted from FFPE tissue blocks using the QIAamp DNA FFPE Tissue Kit (Cat. No. 56404), following the manufacturer’s (QIAGEN, Germany) protocol. The quality and quantity of the isolated DNA were assessed using both NanoDrop spectrophotometry for purity and Qubit fluorometry for accurate concentration measurement. DNA integrity was further confirmed by agarose gel electrophoresis.

Whole-exome libraries were constructed from 50 ng of high-quality genomic DNA using the Twist Bioscience Human Core Exome 2.0 Kit (Cat. No. 104207) manufactured by Twist Bioscience (USA). The library preparation process included DNA fragmentation, end repair, adapter ligation, and sample pooling, followed by targeted exome capture spanning approximately 10–50 Mb of coding regions. Library quality and fragment distribution were validated using the Agilent TapeStation system.

Final libraries were quantified and sequenced on an Illumina NovaSeq 6000 platform using paired-end reads. The sequencing was performed to achieve an average coverage depth of 500X, enabling high-confidence detection of variants within the captured exonic regions.

### 2.3 Sequencing read quality control, preprocessing, and alignment

The quality of the raw sequencing reads was initially assessed using FastQC software (v0.12.1), which provided comprehensive reports on key read quality metrics (25). To enhance the accuracy of downstream analyses, the raw reads underwent preprocessing to remove sequencing adapters and low-quality bases. This critical filtering step, which eliminates potential sequencing artifacts, was performed using Trimmomatic (v0.39) (26). After quality control and trimming, the resulting high-quality reads were aligned to the human reference genome (hg19) using the Burrows-Wheeler Aligner (BWA-MEM) algorithm, a robust tool for mapping short reads to large reference sequences (27). The alignment output, initially in SAM format, was converted to the more efficient BAM format using SAMtools, facilitating optimized storage and seamless integration into subsequent bioinformatics workflows (28). The sequencing data demonstrated high-quality metrics across all samples, with a Q30 score exceeding 90%. Alignment rates were consistently high, surpassing 99%, and the targeted panel coverage was robust, remaining above 95%.

### 2.4 Variant discovery and annotation

Variant discovery followed the GATK Best Practices workflow using GATK v4.3 (29). Somatic variants were identified using the Mutect2 caller with the Twist 2.0 target BED file, and further refined through a series of filtering steps using FilterMutectCalls and Mutect2 filters. These filters removed low-quality calls and germline variants flagged with labels such as contamination, germline, multiallelic, normal_artifact, weak_evidence, panel_of_normals, and clustered_events. Following variant calling, a comprehensive annotation was performed using ANNOVAR, leveraging the RefSeq database for gene-based annotation (30). Public databases, such as COSMIC and ClinVar, along with other resources, were used to annotate the identified variants (31). COSMIC was used to identify previously reported cancer somatic mutations, while ClinVar was leveraged to assess clinical significance and support ACMG/AMP classifications. To distinguish potentially pathogenic mutations from common polymorphisms, population frequency data were integrated from large-scale databases including the 1000 Genomes Project (32), ExAC (33), gnomAD (34), gnomAD-ME (34), and ESP (35). The functional impact of identified missense variants was predicted using multiple in silico tools, including SIFT (36), PolyPhen-2 (37), FATHMM (38), MutationTaster (39), and MutationAssessor (40). Additionally, variants were annotated with CADD scores to estimate their deleteriousness (41). While primarily developed for germline variant interpretation, relevant ACMG/AMP criteria were carefully applied using InterVar (42) to prioritize somatic variants for potential clinical significance within this study. Based on these criteria, all variants were categorized as benign (B), likely benign (LB), likely pathogenic (LP), pathogenic (P), or variants of uncertain significance (VUS).

### 2.5 Somatic variant filtering and classification

To mitigate the inherent challenges of tumor-only analysis, such as distinguishing somatic mutations from germline variants and reducing false positives, stringent bioinformatic filtering strategies were implemented. This approach, while not achieving the same precision as matched tumor-normal sequencing, allowed for the identification of potential somatic alterations in this unique cohort. Somatic variants were filtered and analyzed with a focused interest on a predefined panel based on established cancer genes, largely identified in diverse but often Western cohorts.: *AXIN1*, *BAX*, *BRCA2*, *EGFR*, *ELAVL1*, *KRT18*, *KRT8*, *PIK3R2*, *PRDX1*, *PTEN*, *SLC25A5*, *SOS1*, *TCF7L2*, *TGFB2*, and *TUBB6.* Initial filtering excluded synonymous and intronic variants, retaining only exonic non-synonymous and indel variants predicted to alter protein structure or function. To enrich for clinically and biologically significant alterations, only variants with a minor allele frequency (MAF) ≤1% in population databases (including Middle East population specific gnomAD-ME) were retained, avoiding common polymorphisms.

Variants were annotated as “known” if they were listed in dbSNP, gnomAD, COSMIC, or ClinVar, and labeled as “novel” if no such identifiers were present. dbSNP was primarily utilized to filter out common single nucleotide polymorphisms (SNPs) and other variants likely representing germline polymorphisms, thereby aiding in identifying novel variants not previously reported in the general population. Conversely, COSMIC served as the primary reference for identifying known cancer-associated somatic variants. Functional impact was predicted using multiple in silico tools, including SIFT (36), PolyPhen (37), FATHMM (38), MutationAssessor (40), MutationTaster (39), and CADD (41). Variants were classified as deleterious if they had a CADD score ≥20 and at least three of the other tools also predicted a deleterious effect.

The critical need to identify ‘actionable’ somatic mutations for precision oncology necessitates a standardized pathogenicity assessment approach, similar to germline variants. To improve clinical interpretation, variants were classified as Benign (B), Likely Benign (LB), Pathogenic (P), Likely Pathogenic (LP), or Variants of Uncertain Significance (VUS) using a combination of annotations from ClinVar, InterVar, and consensus in silico predictions. For those lacking ClinVar or InterVar annotations, classification as Deleterious (D) or Neutral (N) was based on CADD scores, Aloft predictions, and VEP impact scores, particularly in the case of indels and splice variants.

For comparative analyses, publicly available colorectal adenocarcinoma data was leveraged from The Cancer Genome Atlas (TCGA-COADREAD) via cBioPortal (accession ID: “coadread_tcga_pan_can_atlas_2018”) (43, 44). To ensure a direct and robust comparison with our Saudi cohort, this large dataset (n = 534) was stringently filtered to include only the identical gene set examined in our study. Furthermore, synonymous, intronic, and common polymorphic variants were excluded, allowing for a focused analysis of potentially pathogenic somatic alterations across both cohorts.

### 2.6 Statistical analysis and data visualization

All statistical analyses were performed using R software version 4.3.2 (45). Data processing and visualization were conducted primarily with the “tidyverse” (46), “ggplot2” (47), and “maftools” (48) packages within the R environment. Tables were generated using the “gt” (49) and “gtsummary” (50) packages for presentation. Specifically, Fisher’s Exact test was used to compare gene mutation frequencies between different groups. To account for multiple comparisons, p-values from these tests were adjusted using the Benjamini-Hochberg method to control the False Discovery Rate (FDR).

## 3 Results

### 3.1 Patient characteristics

A total of 24 patients were recruited for this preliminary study. The cohort predominantly comprised females (n=15, 62.5%), with males accounting for 9 patients (37.5%). The median age at diagnosis was 57 years (Interquartile Range: 42-63 years), with the majority of patients (n=15, 63%) being over 50 years old, while 9 patients were below 50. Tumor staging revealed that 46% of patients had T3 tumors and 42% had T4 tumors, indicating a predominance of locally advanced disease. Regarding lymph node involvement, 46% were classified as N2 and 42% as N3. Metastasis (M1) was observed in 88% of patients, confirming that the majority presented with advanced disease. Only 13% had early-stage cancer without evidence of metastasis. Tumor locations varied across the cohort, with 2 patients (8.3%) having tumors in the Ascending & Transverse colon, 3 (13%) in the Descending colon, 4 (17%) in Multicentric sites, and the largest proportion (n=15, 63%) in the Recto-sigmoid region. Regarding tumor differentiation, 17 patients (71%) had well-differentiated tumors, 4 (17%) had intermediate differentiation, and 3 (13%) had poorly differentiated tumors. Key characteristics of this cohort are further detailed in **Table 1**.

**Table 1:** Clinical characteristics of the patients included in the Saudi cohort.

Characteristic	N = 24*^1^*
Gender	
Female	15 (63%)
Male	9 (38%)
Age (Years)	57 (42 – 63)
Age Group	
<50	9 (38%)
>50	15 (63%)
T Stage	
T 1	3 (13%)
T 3	11 (46%)
T 4	10 (42%)
N Stage	
N1	3 (13%)
N2	11 (46%)
N3	10 (42%)
M Stage	
M0	3 (13%)
M1	21 (88%)
Stage Group	
Early stage	3 (13%)
Advanced stage	21 (88%)
Tumor Site	
Ascending & Transverse colon	2 (8·3%)
Descending colon	3 (13%)
Multicentric	4 (17%)
Recto-sigmoid	15 (63%)
Tumor Differentiation	
Intermediate	4 (17%)
Poor	3 (13%)
Well	17 (71%)

*^1^* n (%); Median (IQR).

The TCGA colorectal patient cohort comprised 64% colon adenocarcinoma, 26% rectal adenocarcinoma, and 10% mucinous adenocarcinoma cases. According to AJCC staging, the majority of patients in this cohort presented with no distant metastasis (M0; 74%), followed by distant metastasis (M1; 12%), undetermined distant metastasis (MX; 10%), and distant metastasis to a single organ or site (M1A; 2%).

### 3.2 Mutational landscape of selected genes

**Figure 1:**
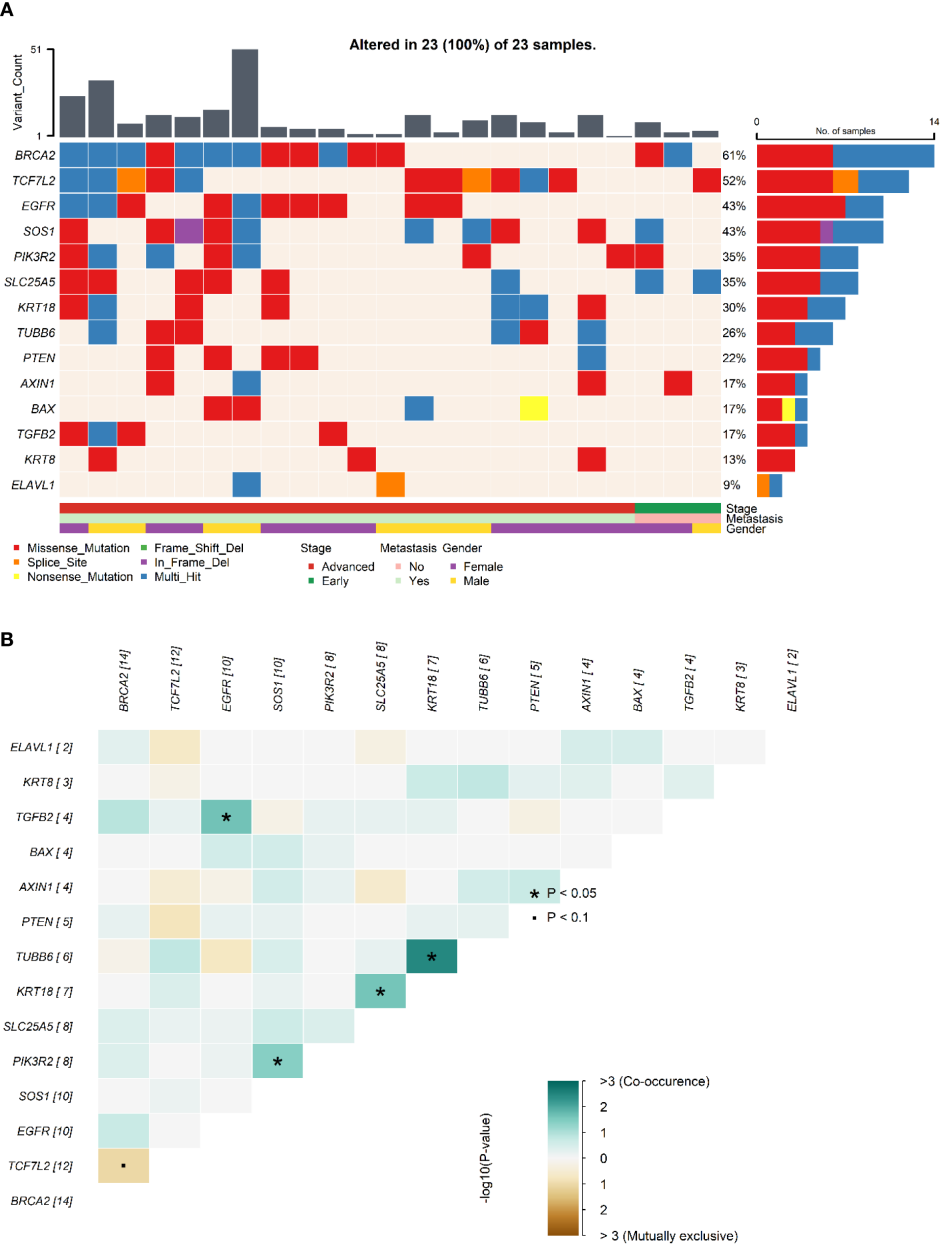
Somatic mutations and gene interaction patterns in Saudi CRC cohort. **(A)**. Oncoplot showing the distribution of somatic mutations in the selected genes across individual samples in the Saudi cohort. Each row represents a gene, and each column represents a patient sample, indicating the presence or absence of mutations. **(B)**. Plot depicting patterns of co-occurring and mutually exclusive gene variants observed across samples in the cohort.* indicates statistical significance (p < 0.05) for co-occurrence or mutual exclusivity. indicates marginal significance (p < 0.1).

Examining the mutational landscape of the selected genes within the cohort revealed that 23 out of the 24 patients harbored at least one somatic variant. The most frequently mutated gene was *BRCA2*, with variants detected in 14 samples (61%). Following closely, the Beta-catenin pathway gene *TCF7L2* was mutated in 52% of patients. Other frequently altered genes included *EGFR* (43%), *SOS1* (43%), *PIK3R2* (35%), and *SLC25A5* (35%). Less frequent mutations were observed in *KRT18* (30%), *TUBB6* (26%), *PTEN* (22%), *AXIN1* (17%), *BAX* (17%), *TGFB2* (17%), *KRT8* (13%), and *ELAVL1* (9%). No mutations associated with *PRDX1* were identified in any of the samples. The distribution of these mutations across individual samples is visually presented in **Figure 1A**. Further analysis of co-mutations identified key patterns of co-occurring and mutually exclusive variants. A statistically significant co-occurrence was noted between *TGFB2* and *EGFR* variants (p < 0.05). Similarly, *TUBB6* variants were found to co-occur significantly with *KRT18*, and *KRT18* with *SLC25A5*, as well as *PIK3R2* with *SOS1*. While not reaching statistical significance, *TCF7L2* variants demonstrated a tendency towards mutual exclusivity with variants in *BRCA2* and *PTEN*. **Figure 1B** depicts the mutation co-occurrence of the selected genes.

### 3.3 Variant characteristics

**Figure 2:**
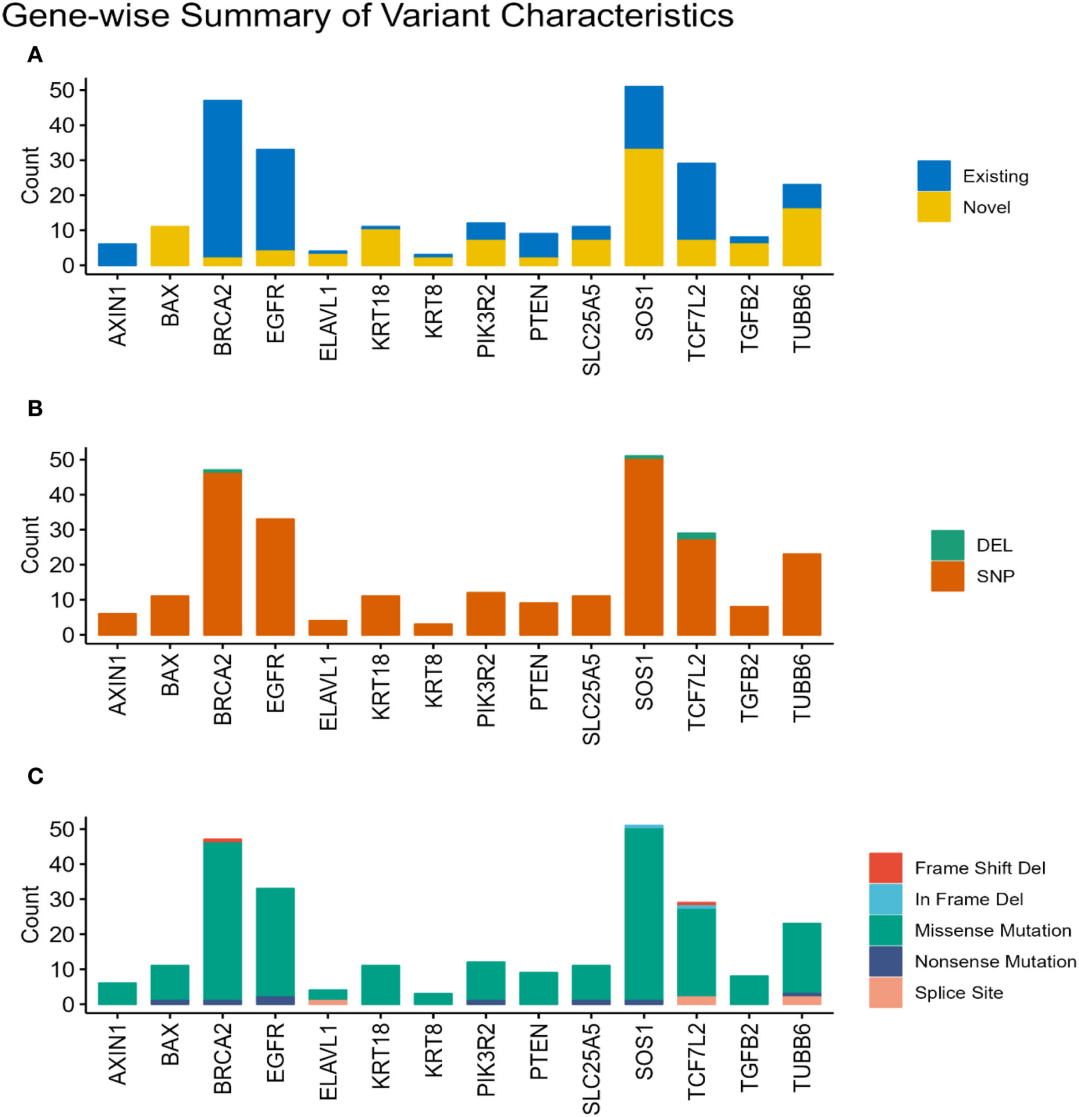
Characterization of somatic variants. Bar plots illustrating the characteristics of the identified somatic variants based on their status **(A)**. (Existing or Novel), **(B)**. type (Indel or SNP), and **(C)** functional classification (e.g., missense, nonsense, frameshift).

Across the 15 selected genes, variants were identified in 14 genes, totaling 258 somatic variants across the cohort. The predominant variant type observed was missense mutations, accounting for the vast majority (n=241, 93%). Nonsense mutations constituted a smaller fraction (n=8, 3%), followed by splice site variants (n=5, 2%). Frameshift and inframe deletions each numbered 2. Overall, 254 variants (98%) were single nucleotide polymorphisms (SNPs), while 4 were insertions/deletions (indels). Among all identified variants across samples, 148 (57%) were classified as existing, meaning they had been previously reported in public databases. Conversely, 110 variants (43%) were considered novel. It is important to note that these counts reflect variant occurrences per sample and may include the same unique variant found in multiple patients. Examining the distribution at the gene level, *SOS1* displayed the highest number of missense mutations (n=49), followed by *BRCA2* (n=45), *EGFR* (n=31), *TCF7L2* (n=25), and *TUBB6* (n=20). For novel variants, the top five genes were *SOS1* (n=33), *TUBB6* (n=16), *BAX* (n=11), *KRT18* (n=10), and *PIK3R2* (n=7). In contrast, existing variants were most frequently found in *BRCA2* (n=45), *EGFR* (n=29), *TCF7L2* (n=22), *SOS1* (n=18), and *PTEN* (n=7). **Figure 2** visually represents these variant characteristics distributed across the genes. The **Figure 2A** illustrates the distribution of variants per gene, categorizing them as existing if they’re present in public databases or novel if they’re not. This distinction highlights the proportion of previously identified mutations versus newly discovered ones. The analysis is further refined in **Figure 2B**, which classifies the variants based on type—either SNPs or Indels (Insertions or Deletions). This classification provides insight into the underlying mutational events, whether they involve a single nucleotide change or a more extensive structural alteration. Finally, **Figure 2C** presents a functional classification of the variants, identifying their potential impact on protein function, such as missense, nonsense, splice site, in-frame deletion, and frameshift deletion mutations, offering a comprehensive view of the molecular consequences of the observed genetic variation.

### 3.4 Differential distribution of somatic mutation: a comparison with TCGA cohort

To understand the uniqueness of the mutational landscape, somatic mutation frequencies of the selected genes in the Saudi cohort were compared with those in the TCGA-COADREAD cohort. This comparison revealed significant differential mutation frequencies across several genes, as detailed in **Table 2**.

**Table 2:** Comparison of gene mutation frequencies between the TCGA-COADREAD cohort and the Saudi cohort, including statistical significance.

Gene	Mutation frequency		p-value*^1^*	Adjusted p-value*^2^*	Significance*^3^*
	TCGA	SAUDI			
AXIN1	3%	17%	7.2 × 10^-3^	9.0 × 10^-3^	**
BAX	1%	17%	2.6 × 10^-4^	4.2 × 10^-4^	***
BRCA2	7%	61%	3.4 × 10^-10^	5.1 × 10^-9^	****
EGFR	3%	43%	2.5 × 10^-9^	1.9 × 10^-8^	****
ELAVL1	1%	9%	5.0 × 10^-2^	5.4 × 10^-2^	ns
KRT18	1%	30%	2.4 × 10^-7^	6.0 × 10^-7^	****
KRT8	1%	13%	4.5 × 10^-3^	6.5 × 10^-3^	**
PIK3R2	2%	35%	8.3 × 10^-8^	2.5 × 10^-7^	****
PRDX1	1%	0%	1.0	1.0	ns
PTEN	6%	22%	1.8 × 10^-2^	2.1 × 10^-2^	*
SLC25A5	1%	35%	6.3 × 10^-9^	3.2 × 10^-8^	****
SOS1	4%	43%	6.9 × 10^-8^	2.5 × 10^-7^	****
TCF7L2	11%	52%	3.1 × 10^-6^	5.9 × 10^-6^	****
TGFB2	3%	17%	4.8 × 10^-3^	6.5 × 10^-3^	**
TUBB6	1%	26%	1.1 × 10^-6^	2.3 × 10^-6^	****

*^1^* Fisher’s exact test.

*^2^* Benjamini-Hochberg FDR.

*^3^* ns: P > 0.05, *: P ≤ 0.05, **: P ≤ 0.01, ***: P ≤ 0.001, ****: P ≤ 0.0001.

**Figure 3:**
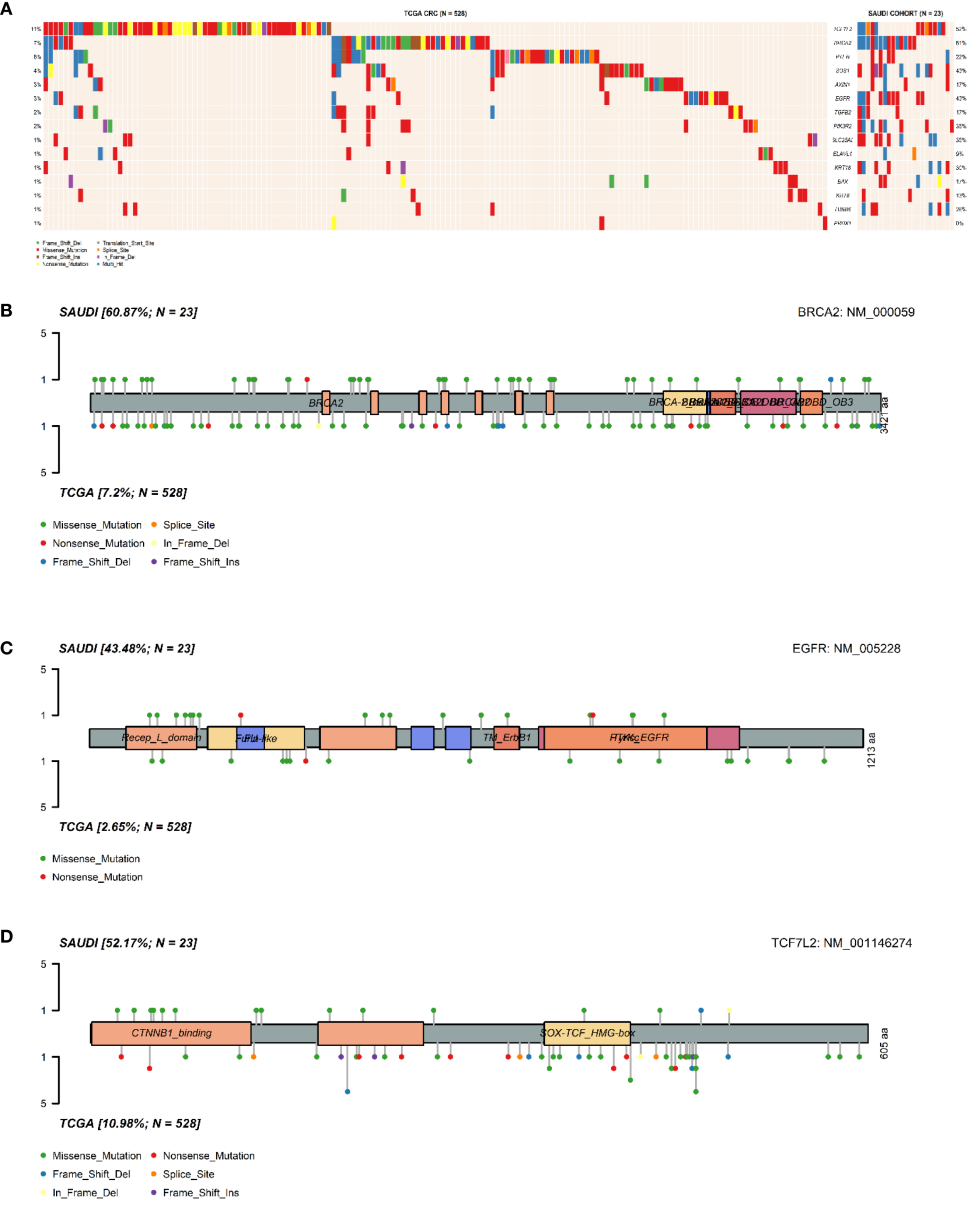
Comparative analysis of somatic mutations in Saudi and TCGA CRC cohorts. **(A)**. Oncoplot comparing the distribution and frequency of mutations in the selected genes between the Saudi cohort and the TCGA-COADREAD cohort. Lolliplot illustrating the distribution of **(B)**. BRCA2 variants, **(C)**. EGFR variants, **(D)**. TCF7L2 variants across protein domains, comparing patterns observed in the TCGA cohort and the Saudi cohort.

**Figure 4:**
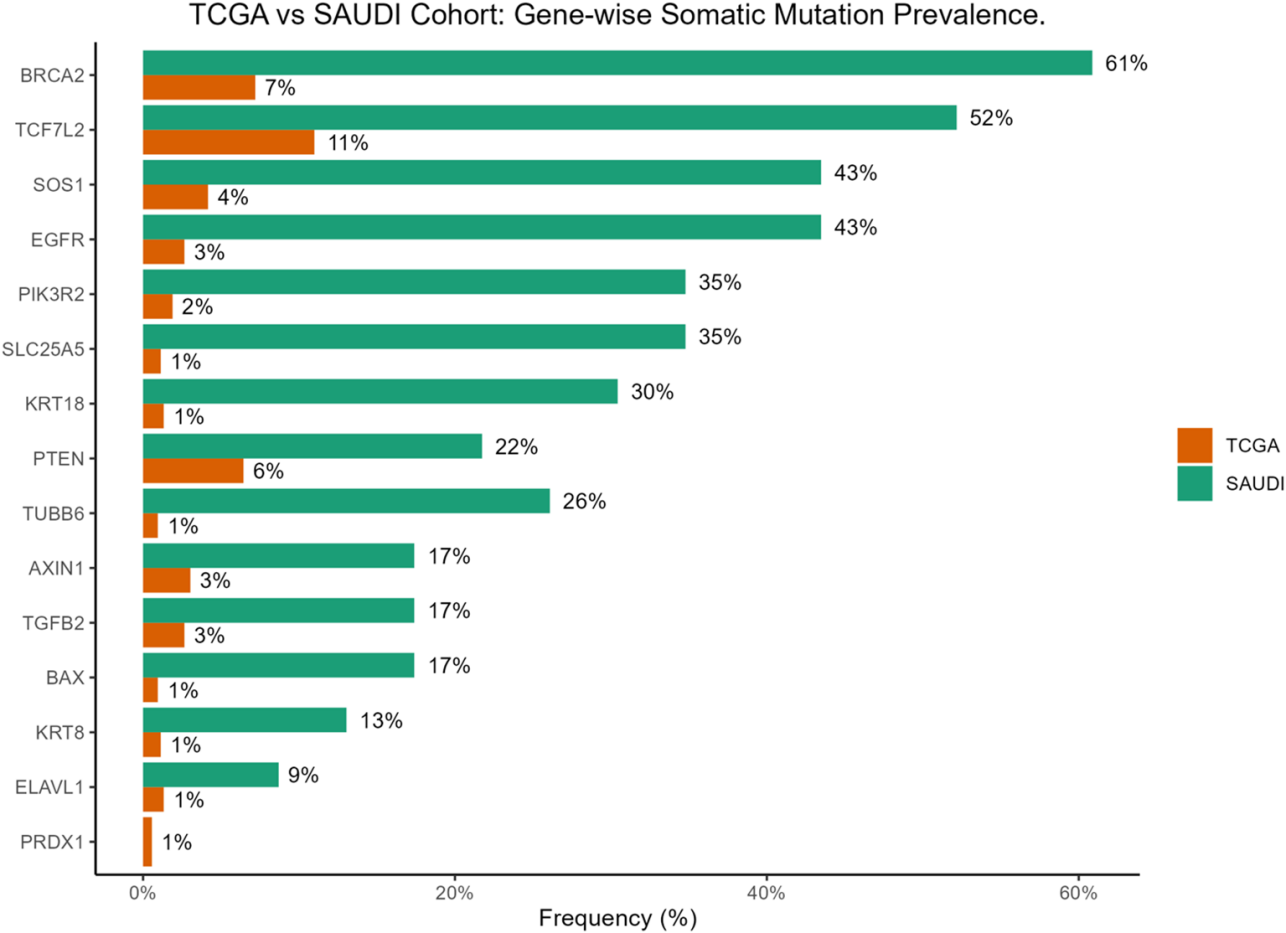
Bar plot comparing gene-wise somatic mutation prevalence between the TCGA-COADREAD and the Saudi cohorts.

Notably, *BRCA2* mutations were significantly enriched in the Saudi cohort compared to the TCGA cohort (61% vs 7%, p = 3.4×10^−10^). Similar significant enrichments in the Saudi cohort were observed for *EGFR* (43% vs 3%, p = 2.5×10^−9^), *SLC25A5* (35% vs 1%, p = 6.3×10^−9^), *SOS1* (43% vs 4%, p = 6.9×10^−8^), *PIK3R2* (35% vs 2%, p = 8.3×10^−8^), *KRT18* (30% vs 1%, p = 2.4×10^−7^), *TUBB6* (26% vs 1%, p = 1.1×10^−6^), and *TCF7L2* (52% vs 11%, p = 3.1×10^−6^). It is important to note that our cohort’s relatively small sample size (n=24) may contribute to these seemingly inflated frequencies, making rare events appear disproportionately common compared to large, heterogeneous datasets like TCGA. Therefore, these figures should be interpreted as observed patterns within our specific cohort rather than definitive population prevalences. These differential mutation patterns are visually presented in **Figure 3A** and **Figure 4**. Further investigation into the distribution of variants within individual genes did not reveal a universal pattern of specific domain enrichment. For *BRCA2* and *EGFR*, the variant distribution across protein domains appeared similar in both cohorts. However, a contrasting pattern was observed for *TCF7L2*, where variants in the Saudi cohort were more enriched in the CTNNB1 binding domain, whereas those in the TCGA cohort showed greater enrichment in the SOX-TCF-HMGBOX domain. The domain-level distribution of variants for these genes in each cohort is depicted in **Figures 3B–D**.

### 3.5 Pathogenic variants identified in the cohort

Based on classification using ClinVar, InterVar, and functional prediction tools, a total of 25 variants were identified as Pathogenic (P), Likely Pathogenic (LP), or Deleterious (D) within the cohort. These potentially impactful variants were distributed across nine genes: *EGFR* (n=7), *TCF7L2* (n=5), *PIK3R2* (n=3), *TGFB2* (n=3), *BRCA2* (n=2), *SOS1* (n=2), *ELAVL1* (n=1), *PTEN* (n=1), and *TUBB6* (n=1). The types of variants categorized as P/LP or D included 13 missense mutations, 6 nonsense mutations, 4 splice site variants, 1 frameshift deletion, and 1 in-frame deletion. Among these, 13 variants were novel, not previously reported in public databases, and were classified within this category. These novel pathogenic/deleterious variants included specific alterations in *EGFR* (NM_001346941.2:c.C2365T:p.Q789X), a splice variant in *ELAVL1*, multiple variants in *PIK3R2* (NM_005027.4:c.C679T:p.Q227X; NM_005027.4:c.C374T:p.P125L; NM_005027.4:c.G415A:p.G139R), variants in *SOS1* (NM_001382394.1:c.G2267A:p.W756X; NM_001382394.1:c.249_251del:p.Q84del), and several variants in *TCF7L2* (Splice Variant; NM_001146285.1:c.1413_1435del:p.N475Rfs*7;NM_001349870.2:c.G101T:p.W34L; NM_001146285.1:c.1411_1412delinsTT:p.P471F), as well as a missense mutation in *TGFB2* (NM_003238.6:c.G907A:p.A303T) and a splice variant in *TUBB6*. Detailed information on each of these pathogenic or deleterious variants is provided in **Table 3**.

**Table 3:** List of Pathogenic (P), Likely Pathogenic (LP), and Deleterious (D) variants identified in the Saudi cohort, with associated details.

Gene	Variant	Variant class	Variant ID	Variant status	*In silico* prediction*^1^*	ClinVar status*^1^*	ACMG classification (InterVar)*^1^*	Interpreted classification*^1^*
*BRCA2*	NM 000059.4:c.C2809T:p.Q937X	Nonsense	rs2137490179, COSV66458068	Existing	D	P	P	P
*BRCA2*	NM 000059.4:c.C7867T:p.H2623Y	Missense	rs1566244783	Existing	D	LP	VUS	LP
*EGFR*	NM 001346941.2:c.G485A:p.R162K	Missense	rs2128939760	Existing	D	–	LP	LP
*EGFR*	NM 001346941.2:c.C709T:p.Q237X	Nonsense	rs1334180707	Existing	D	–	P	P
*EGFR*	NM 001346941.2:c.A1660T:p.I554F	Missense	rs1786873149	Existing	D	–	LP	LP
*EGFR*	NM 001346941.2:c.C2365T:p.Q789X	Nonsense	–	Novel	D	–	P	P
*EGFR*	NM 001346941.2:c.G1375A:p.V459M	Missense	rs483352805	Existing	D	P	P	P
*EGFR*	NM 001346941.2:c.G406A:p.G136R	Missense	rs2128938801	Existing	D	–	LP	LP
*EGFR*	NM 001346897.2:c.G473A:p.G158E	Missense	rs2128932598, COSV51777649	Existing	D	–	LP	LP
*ELAVL1*	-	Splice Site	–	Novel	D	–	–	D
*PIK3R2*	NM 005027.4:c.C679T:p.Q227X	Nonsense	–	Novel	D	–	P	P
*PIK3R2*	NM 005027.4:c.C374T:p.P125L	Missense	–	Novel	D	–	LP	LP
*PIK3R2*	NM 005027.4:c.G415A:p.G139R	Missense	–	Novel	D	–	LP	LP
*PTEN*	NM 000314.8:c.C112T:p.P38S	Missense	rs587780004, CM1617835, COSV100911304, COSV64289791	Existing	D	LP	VUS	LP
*SOS1*	NM 001382394.1:c.G2267A:p.W756X	Nonsense	–	Novel	D	–	P	P
*SOS1*	NM 001382394.1:c.249 251del:p.Q84del	In Frame Del	–	Novel	D	–	–	D
*TCF7L2*	-	Splice Site	COSV53344934, COSV53351975	Existing	D	–	–	D
*TCF7L2*	-	Splice Site	–	Novel	D	–	–	D
*TCF7L2*	NM 001146285.1:c.1413 1435del:p.N475Rfs*7	Frame Shift Del	–	Novel	D	–	–	D
*TCF7L2*	NM 001349870.2:c.G101T:p.W34L	Missense	–	Novel	D	–	–	D
*TCF7L2*	NM 001146285.1:c.1411 1412delinsTT:p.P471F	Missense	–	Novel	D	–	–	D
*TGFB2*	NM 003238.6:c.G907A:p.A303T	Missense	–	Novel	D	–	LP	LP
*TGFB2*	NM 003238.6:c.G932A:p.R311K	Missense	rs1064793278	Existing	D	LP	LP	LP
*TGFB2*	NM 003238.6:c.G630A:p.W210X	Nonsense	COSV100860033	Existing	D	–	P	P
*TUBB6*	-	Splice Site	–	Novel	D	–	–	D

^1^D, Deleterious; N, Neutral; P, Pathogenic; LP, Likely Pathogenic; VUS, Variant of Unknown Significance.

### 3.6 Genomic-based pathway alteration in the cohort

**Figure 5:**
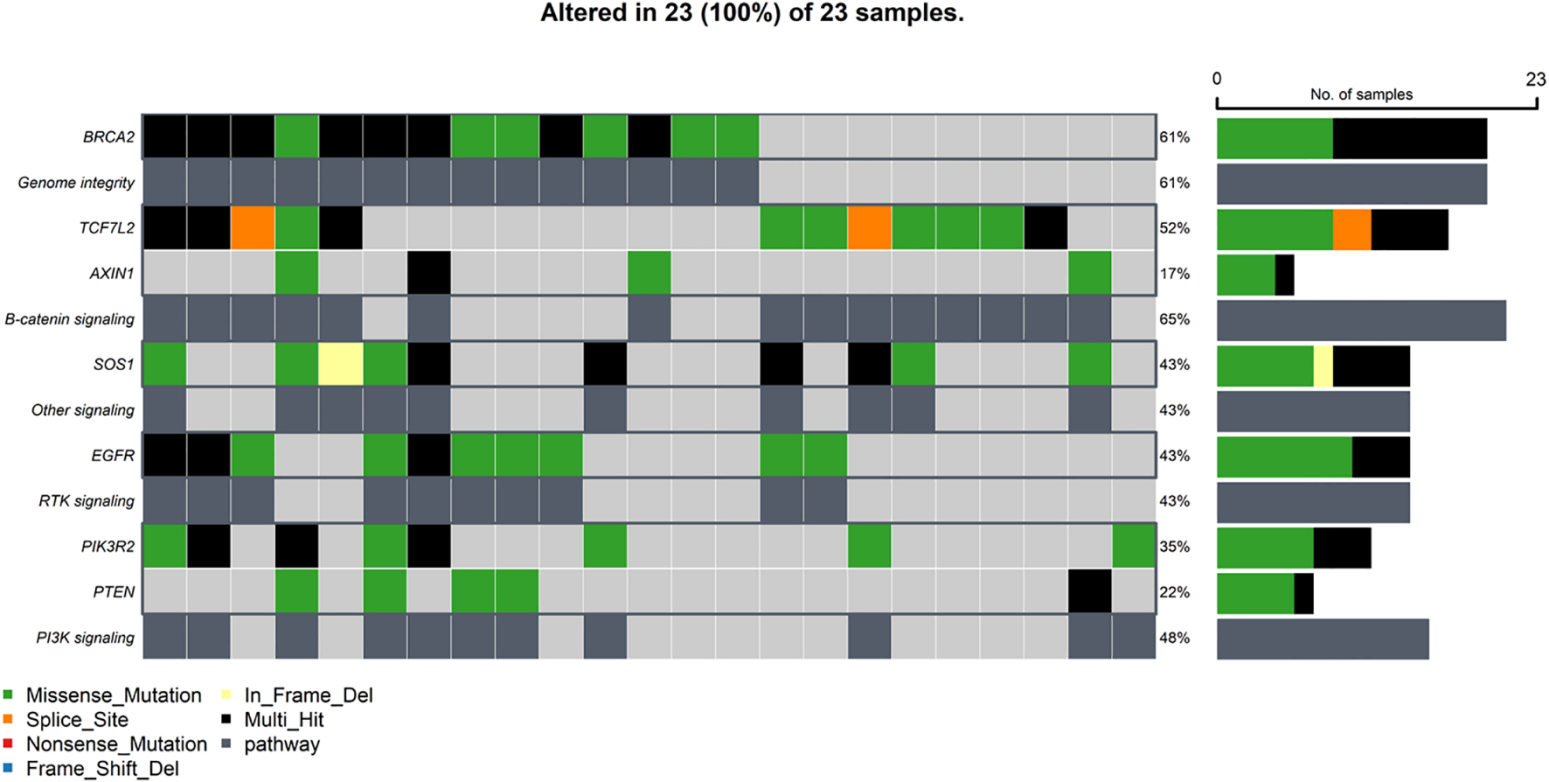
Oncoplot depicting the distribution of alterations in top genomic-based pathways across samples in the Saudi cohort.

Building upon the observed somatic mutations, this study investigated the functional impact of these alterations by assessing the disruption of key cellular pathways (**Figure 5**). The analysis, based on the frequency of mutations in the selected genes, revealed widespread pathway alterations within the cohort. The Genomic Integrity pathway, specifically the Homologous Recombination Repair (HRR) mechanism, was found to be altered in a substantial proportion of patients (61%), primarily due to mutations identified in the *BRCA2* gene. The critical Beta-catenin signaling pathway was disrupted in an even larger percentage of cases (65%), driven by mutations in both *TCF7L2* and *AXIN1*. Downstream signaling cascades were also frequently affected; the PI3K signaling pathway showed alterations in 48% of patients, with the pathway being driven by a PIK3R2 mutation alone in six patients, a PTEN mutation in five patients, and mutations in both genes in two patients. Furthermore, Receptor Tyrosine Kinase (RTK) signaling, specifically involving the EGFR pathway, was altered in 43% of cases due to *EGFR* mutations. Another related signaling cascade, likely the RAS pathway, was also found to be altered in 43% of patients, attributable to mutations in the *SOS1* gene.

## 4 Discussion

Our preliminary study offers an initial exploration of colorectal cancer within a Saudi Arabian patient cohort. By employing whole-exome sequencing, we’ve begun to examine a selected panel of cancer-associated genes. This targeted approach provides early insights into the mutation frequencies, pathogenic variants, and pathway disruptions that appear to be specific to this population whole-exome. This approach reveals molecular features that differ from international datasets, offering valuable information for precision oncology efforts in this region. Previously, we identified several variants associated with breast cancer (51) and iron deficiency (52) in the Saudi population.

Our cohort (**Table 1**) had a median age of diagnosis of 57 years, slightly younger than the global average, with 38% of patients diagnosed before age 50, aligning with global trends of rising early-onset CRC (53, 54). Alarmingly, 88% of patients presented with advanced-stage disease, including metastatic cases, suggesting delays in diagnosis and potentially limited access to early screening programs. This reinforces the urgent need to establish or enhance national CRC screening initiatives in Saudi Arabia (55).

Genomic analysis revealed a distinct mutational profile compared to the TCGA-COADREAD cohort (**Table 2**; **Figures 3**, **4**). Among the most striking findings was the significantly higher frequency of *BRCA2* mutations (61% vs. 7%), a gene canonically associated with DNA repair through homologous recombination (56–58). This observation is consistent with a recent NGS-based study of Saudi CRC patients, which reported *BRCA2* mutations in 79% of cases (2), further supporting the potential significance of this gene in the regional disease profile. This notable enrichment may reflect population-specific genomic signatures or underlying hereditary predispositions. *BRCA2* mutations were also a major contributor to the disruption of the Genomic Integrity pathway in over 60% of patients (**Figure 5**), indicating to defective DNA repair as a possible hallmark of CRC in this population. The high metastatic rate observed in our Saudi cohort (M1 in 88% of cases, compared to 22% in TCGA) could potentially be linked to these distinct mutational patterns, particularly alterations in genes like BRCA2, which are implicated in genomic instability and may promote more aggressive tumor behavior and metastatic progression.

Similarly, *EGFR* (43% vs. 3%) and *TCF7L2* (52% vs. 11%) were more frequently mutated, both genes central to key oncogenic signalling cascades. EGFR is a well-established therapeutic target in CRC, particularly in KRAS wild-type tumors, and its high mutation rate may open avenues for targeted therapies such as tyrosine kinase inhibitors (59). It is important to clarify that EGFR expression, rather than EGFR mutations, serves as the key clinical biomarker for guiding anti-EGFR therapy in CRC. While EGFR mutations were observed in our study, they do not typically serve the same predictive role for response to these therapies as EGFR expression, especially in the context of RAS/BRAF wild-type tumors. As this study focused on genomic alterations, EGFR expression was not assessed.*TCF7L2*, a transcription factor in the Wnt/β-catenin pathway, showed not only a higher frequency of mutations but also a unique domain-level mutation distribution, with a preference for the CTNNB1-binding domain in the Saudi cohort (**Figure 3D**). This observation may suggest altered transcriptional regulation of Wnt target genes, contributing to uncontrolled proliferation (60, 61). Additionally, genes such as *SOS1*, *PIK3R2*, and *SLC25A5* were frequently mutated, with *SOS1* mutations contributing to both RTK and RAS pathway alterations in 43% of patients (**Figure 5**). The PI3K/AKT/mTOR pathway was also disrupted in 48% of cases (**Figure 5**), consistent with findings in aggressive or chemoresistant CRC subtypes (62, 63). Together, these results highlight the merging of multiple signalling pathways that may drive tumorigenesis through redundant or synergistic mechanisms in this population.

Notably, 25 pathogenic or likely pathogenic variants were identified and classified, including 13 novel variants not cataloged in public databases (**Table 3**), reinforcing the genetic uniqueness of the Saudi population and the importance of diverse representation in cancer genomics (64). Particularly, deleterious *EGFR* and *BRCA2* variants suggest therapeutic vulnerability, as tumors harboring these mutations may be particularly susceptible to *EGFR*-targeted therapies and PARP inhibitors, respectively, due to their reliance on dysregulated signalling pathways or impaired DNA repair mechanisms (65, 66). Further, novel *TCF7L2* and *TGFB2* mutations may represent as-yet-undefined drivers of tumor progression. Notably, *EGFR* harbored the largest number of pathogenic variants, including several nonsense and missense mutations. Among them, NM_001346941.2:c.C2365T:p.Q789X, a novel truncating mutation, which may lead to loss of function or altered receptor dynamics, with potential implications for treatment resistance. Similarly, deleterious splice site and nonsense mutations in *TCF7L2* and *PIK3R2* may result in disrupted protein function, demonstrating a need for further experimental validation and possibly functional annotation through CRISPR-based models or transcriptome analysis.

Comparative analysis revealed unique mutational patterns in the Saudi cohort (**Table 2**; **Figures 3**, **4**), including high mutation frequencies in *KRT18* (30% vs. 1%) and *TUBB6* (26% vs. 1%). While these genes are less well-characterized in CRC, their frequent mutation suggests a potential role in cytoskeletal regulation and cellular adhesion, processes critical to metastasis and tumor invasion (16, 67). Further, *TUBB6* encodes a beta-tubulin isotype that is a critical component of the microtubule cytoskeleton, supporting cell division and intracellular transport (18). Disruptions in tubulin function may lead to defective cell division and contribute to chromosomal instability (CIN), a key factor in CRC progression. In contrast, the absence of *PRDX1* mutations despite their presence in TCGA may reflect distinct selective pressures. Mutual exclusivity between *TCF7L2* and *BRCA2*/*PTEN* mutations (**Figure 1**) further illustrates the complex interplay between tumor suppressors and signaling regulators, and points to potential functional redundancy or antagonism in tumor suppressor networks. Further mechanistic research is needed to understand how these genes interact. A crucial limitation of this study is the absence of functional assays to validate the impact of the identified mutations. While we have pinpointed specific pathogenic variants in different genes, our findings are based solely on genomic sequencing data and in-silico tools. The identification of these variants, however, provides a strong basis for future functional research to confirm their exact roles in CRC pathogenesis.

Our exploratory study reveals distinct mutational frequencies in the genes examined, yet a striking concordance with The Cancer Genome Atlas (TCGA) pan-cancer pathway analysis emerges upon converging these mutations onto their respective signaling networks. This suggests that while individual genes may exhibit varying mutation rates, the overall disruption of key oncogenic pathways remains remarkably consistent across different patient cohorts. Specifically, our cohort’s RTK-RAS signaling pathway was found to be altered in 69% of patients, primarily driven by mutations in EGFR or SOS1. This figure aligns closely with the TCGA’s findings, which report RTK-RAS pathway alterations in a wide range of colorectal cancer (CRC) subtypes, from 66% to 99% in chromosomal instability (CRC-CIN), genomically stable (CRC-GS), and microsatellite instability (CRC-MSI-POLE) subtypes (68). Similarly, the PI3K signaling pathway was altered in 48% of our patients, a frequency comparable to the 32–68% range observed in the TCGA cohort (68). This consistency highlights the central and conserved role of these pathways in colorectal carcinogenesis, regardless of the specific mutational drivers. In contrast, a notable divergence was observed in the Homologous Recombination Repair (HRR) pathway. Our cohort showed alterations in 61% of cases, a stark difference from the 21% reported in the TCGA CRC cohort (69). This discrepancy could reflect differences in the patient demographics, environmental exposures, or specific genetic backgrounds of our study population compared to the broader, more geographically diverse TCGA cohort. Alternatively, it might point to a unique, more frequent HRR deficiency in our patient group, which could have significant implications for therapeutic response to agents like PARP inhibitors. Further investigation is warranted to understand the factors underlying this significant variation and its clinical relevance.

While this exploratory study offers valuable initial insights into the molecular landscape of colorectal cancer in a Saudi Arabian cohort, several limitations warrant acknowledgment. Primarily, the small sample size significantly impacts the statistical power and generalizability of our findings. This restricted cohort also meant that the potential influence of confounding factors such as age, sex, and comorbidities on the observed molecular features could not be deeply explored; however, these will be critical considerations for larger-scale future investigations. Given that nine patients were diagnosed before the age of 50, the possibility of hereditary CRC cannot be excluded; however, as our study employed tumor-only sequencing without germline analysis, hereditary contributions could not be specifically addressed.

Furthermore, the reliance on a pre-defined gene panel for whole-exome sequencing, while enabling a targeted analysis, might introduce a degree of selection bias. This approach inherently limits the discovery of novel or population-specific driver mutations that fall outside the selected genes. To overcome this, future studies should consider employing broader sequencing strategies, such as whole-exome sequencing without a targeted panel or whole-genome sequencing, to provide a more comprehensive and unbiased view of the genomic landscape.

Another significant limitation arises when comparing our small cohort to large, ethnically diverse, yet predominantly European, datasets like The Cancer Genome Atlas (TCGA). These comparisons should be interpreted as preliminary, given the substantial differences in cohort size and ethnic composition. Moreover, methodological discrepancies in variant calling pipelines present a challenge for direct comparisons. Our study utilized a tumor-only whole-exome sequencing approach with a single variant caller (Mutect2), whereas large-scale initiatives like TCGA often employ matched tumor-normal sequencing and may integrate results from multiple variant callers. This difference in methodology can influence observed mutation frequencies and should be carefully considered when interpreting cross-dataset comparisons. Despite these limitations, our preliminary findings highlight the unique molecular features within this population, underscoring the necessity for further, more extensive, and methodologically harmonized research to advance precision oncology in Saudi Arabia.

## 5 Conclusion

Together, these initial findings underline the heterogeneity of CRC and the limitations of extrapolating Western-derived genomic data to other populations (70). The high frequency of actionable and novel mutations supports the implementation of population-specific precision oncology strategies. Expanding sequencing efforts and developing regional variant databases are essential steps toward integrating genomic data into clinical practice in Saudi Arabia and the broader Middle East. Future larger cohort studies are warranted to further validate and expand upon these initial findings.

## Data Availability

The datasets presented in this study can be found in online repositories. The names of the repository/repositories and accession number(s) can be found below: https://www.ncbi.nlm.nih.gov/, PRJNA1149347.
